# Discrimination of Spanish-Style Green Olives Inoculated with Undesirable Microbiota Using E-Nose, Chemometrics and Volatile Compound Profiles

**DOI:** 10.3390/foods15050934

**Published:** 2026-03-06

**Authors:** Daniel Martín-Vertedor, Chunyu Tian, Jesús Lozano, Olga Monago-Maraña, Fabricio Chiappini, Francisco Pérez-Nevado

**Affiliations:** 1 Aquaculture Center ‘Las Vegas del Guadiana’, Regional Government of Extremadura, N-5, km 391.7, Villafranco del Guadiana, 06195 Badajoz, Spain; 2 Research Institute of Agricultural Resources (INURA), University of Extremadura, Avda de la Investigación, s/n, 06006 Badajoz, Spain; jesuslozano@unex.es (J.L.); fpen@unex.es (F.P.-N.); 3 Faculty of Biological and Chemical Engineering, Chongqing University of Education, Chongqing 400067, China; tchunyu0329@outlook.com; 4 Industrial Engineering School, University of Extremadura, 06006 Badajoz, Spain; 5 Department of Analytical Sciences, Faculty of Sciences, Universidad Nacional de Educación a Distancia (UNED), 28040 Madrid, Spain; olgamonago@ccia.uned.es; 6 Departamento de Matemática, Facultad de Bioquímica y Ciencias Biológicas, Universidad Nacional del Litoral, Ciudad Universitaria, Paraje El Pozo s/n, Santa Fe 3000, Argentina; fabriciochiappini@gmail.com; 7 Área de Nutrición y Bromatología, Departamento de Producción Animal y Ciencia de los Alimentos, Escuela de Ingenierías Agrarias, Universidad de Extremadura, Avda. Adolfo Suárez s/n, 06007 Badajoz, Spain

**Keywords:** table olives, aroma, e-nose, contaminant microorganisms, chemometric analysis

## Abstract

This study evaluated the potential of electronic nose (E-nose) technology to discriminate Spanish-style green table olives spoiled by different bacterial strains. Microbial growth, physicochemical properties, sensory attributes, and volatile organic compounds (VOCs) profiles were analyzed to assess spoilage patterns. The results indicated strain-dependent microbial survival during incubation, with *Bacillus cereus* and *Enterobacter cloacae* showing the highest tolerance. Inoculated olives exhibited significant changes in color, texture, pH, phenolic content, and antioxidant activity compared to the Control. Sensory evaluation revealed a reduction in positive attributes and the emergence of defects such as cooked, rancid, and woody aromas, particularly in olives inoculated with *B. cereus* and *Escherichia coli*. VOC analysis confirmed these alterations, showing strain-specific increases in aldehydes, phenols, and esters, along with reductions in alcohols and acids. Principal component analysis (PCA) of E-nose data successfully distinguished two groups—spoiled and non-spoiled samples—explaining 84.8% of variance, while Partial Least Squares Discriminant Analysis (PLS-DA) achieved a classification accuracy of 90.4%. These findings highlight the E-nose as a rapid, non-destructive, and reliable tool for detecting bacterial spoilage in table olives, with potential applications in quality control and early spoilage detection.

## 1. Introduction

Table olives are recognized as a key plant-derived fermented product in the Mediterranean region, playing a central role in both agriculture and food processing [1]. Spain is a leading producer, with 854,000 tons of table olives being produced in the 2023/24 crop year, and Spain is one of the world’s top exporters of table olives [2]. As a major agri-food export, table olives generate added value for farmers and contribute positively to Spain’s trade balance. According to data from the International Olive Council (IOC), global production in the provisional 2023/24 crop year is estimated at 2,828,500 tons. The global table olives market was valued at approximately US $4.2 million in 2023 and is projected to reach around US $5.7 million by 2032, reflecting a compound annual growth rate (CAGR) of about 3.3% between 2024 and 2032, driven by increasing consumer interest in healthy eating and Mediterranean-style diets [3].

Table olives are mostly processed using the “Spanish-style” method. In this process, olives are treated with a NaOH solution to remove fruit bitterness, followed by a water washing step and subsequent immersion in brine, in which a spontaneous fermentation process typically occurs [4]. The fermentation is primarily driven by yeasts and lactic acid bacteria [5], which impart the characteristic aroma and flavor to table olives [6]. Nevertheless, several critical stages in this process may lead to abnormal fermentation and the development of defects in the final product. Therefore, strict monitoring of brine chemical parameters (e.g., pH, acidity) during fermentation is essential to ensure product quality [7]. When conditions are unfavorable, alterations are mainly caused by bacteria. The presence of microorganisms during fermentation—including bacteria such as *Enterobacteriaceae*, *Clostridium*, and *Propionibacteriaceae*, as well as fungi such as *Aspergillus* and *Penicillium*—leads to product deterioration and the development of undesirable organoleptic characteristics [6,8].

The main off-odors found in Spanish-style table olives include Zapateria (sweaty/leathery), butyric (rancid butter), putrid, musty, rancid, and vinegary aromas. These unpleasant odors result from microbial spoilage, which leads to the formation of specific VOCs responsible for each defect. Zapateria is mainly associated with butanoic acid, (E)-3-hexenoic acid, hexanoic acid, and pentanoic acid, while butyric aroma is linked to butanoic acid, pentanoic acid, propanoic acid, and butan-2-ol. Putrid defects are associated with isopropyl alcohol, phenylethyl alcohol, propanoic acid, 2,4-dimethyl-heptane, and 3-methyl-1-butanol, whereas musty odors are primarily due to 2-methoxy-phenol, 2,4-dimethyl-heptane, and styrene [9]. Thus, these VOC can be considered chemical markers for identifying and characterizing each sensory defect during quality assessment.

Traditional methods for analyzing the volatile compounds in fermented olives mainly rely on chromatographic techniques and sensory evaluation. Currently, quality defects in table olives are detected using analytical tools such as gas chromatography (GC), high-performance liquid chromatography (HPLC), gas chromatography–mass spectrometry (GC-MS), mass spectrometry (MS), ion mobility spectrometry (IMS), and infrared spectrometry (IR). However, these techniques are often limited by their complexity, high cost, specialized equipment requirements, and time-consuming procedures [10].

The primary method for sensory analysis, which is also the official approach according to International Olive Council (IOC) regulations, involves selecting, training, and developing the skills of a panel of 8–10 tasters to accurately assess the severity of defects in table olives. This method establishes the criteria and procedure for the sensory evaluation of the odor, taste, texture, and commercial classification of table olives. Furthermore, olives are categorized into different classes based on their sensory characteristics [5]. However, despite being the reference method, sensory panel evaluation requires substantial training of panelists and considerable equipment to perform comprehensive analyses [11,12]. According to the IOC standards [5], the classification of brines was determined by the predominant defect perception (PDP) intensity, as described below: Extra or Fancy (PDP ≤ 3); First, 1st, Choice, or Select (3 < PDP ≤ 4.5); Second, 2nd, or Standard (4.5 < PDP ≤ 7.0); and olives unsuitable for sale as table olives (PDP > 7).

For these reasons, there is a clear need for more precise and faster analytical tools to detect and quantify aromatic compounds in table olives, thereby ensuring the production of high-quality products. As a cutting-edge technique, the ultra-fast gas chromatography electronic nose (GC E-nose) has been used to identify key compounds, including 2-nonanone, cyclodecanol, eugenol, and 1,3-cyclooctadiene in Chinese carps [13]. Furthermore, this technology has been applied to the identification of volatile differential markers for Baijiu—a traditional Chinese distilled spirit—demonstrating its potential as a valuable tool for food authenticity assessment and quality control [14].

Furthermore, in recent years, novel techniques for the analysis of aromatic compounds in foods have emerged. Among these methods, the electronic nose (E-nose) stands out for its ability to effectively characterize the aroma profile of food samples. The E-nose primarily consists of two systems: a detection or chemical transduction system, which is used to detect volatile compounds in food, and an automated pattern recognition system, which is designed to process and interpret the data. A key component of the E-nose system is its sensor array, which is responsible for recognizing different aromatic compounds. As an efficient, low-cost, fast, non-destructive, and promising technology, E-nose has been successfully applied in a wide range of applications in the food industry, including freshness assessment, process monitoring, flavor evaluation, authenticity determination, quality control, origin tracing, and pesticide residue detection [15].

Moreover, these electronic devices are also employed to detect a variety of defects in food matrices caused by contaminating microorganisms, such as *Penicillium expansum* in apples, *Botrytis cinerea* in tomatoes, *Galactomyces*, *Penicillium*, *Aspergillus* and *Fusarium* in Spanish-style table olives [16,17,18,19]. E-noses are widely used in table olive research. For instance, two olive cultivars were evaluated using an E-nose to detect acrylamide, a carcinogenic compound formed during the sterilization process of Californian-style black olives [20,21]. Additionally, E-noses have proven effective in distinguishing spoiled table olives based on their VOC profiles. The high sensitivity of the E-nose was further demonstrated in studies on Spanish-style olives inoculated with spoilage molds, where it was able to detect multiple strains of the same mold species [22,23].

Despite these advantages, the use of E-nose systems also presents several limitations. One of the main drawbacks is the lack of specificity of the sensors, which respond to groups of compounds rather than to individual VOCs, making it difficult to achieve precise chemical identification. In addition, sensor drift over time, sensitivity to environmental factors such as humidity and temperature, and the need for frequent calibration can affect measurement reliability and reproducibility. Another important limitation is the strong dependence on data processing and chemometric models, which require large, well-designed training datasets and may not be easily transferable between different applications or matrices. Consequently, E-nose devices are often used as complementary tools rather than as standalone analytical techniques, and their results are usually validated using conventional methods such as gas chromatography–mass spectrometry (GC–MS).

Given the limitations of traditional analytical methods—such as high costs, complexity, and long processing times—it is essential to develop and implement more efficient, accurate, and rapid technologies for detecting and quantifying aromatic compounds in table olives. Improved analytical tools would not only enhance the monitoring of quality and early detection of defects but also support producers in maintaining consistent flavor profiles and meeting high consumer expectations. Ultimately, such advancements are crucial for ensuring the production of premium-quality olives and strengthening competitiveness in the market. Accordingly, this study aimed to evaluate the effectiveness of an E-nose system in distinguishing Spanish-style table olives affected by bacterial spoilage, as well as to assess its quantitative capability in evaluating various sensory attributes.

## 2. Materials and Methods

### 2.1. Elaboration of Spanish-Style Green Table Olives and Bacterial Contamination

‘Carrasqueña’ olives at the green stage of maturation were processed Spanish-style throughout this study by a company located in the southwest of Extremadura, Spain. Olives were placed in fermenters (236 L of total capacity) and treated with sodium hydroxide to remove their bitterness. The olives were then washed twice to eliminate excess NaOH, and 10% brine was added, initiating natural fermentation, which proceeded for 3 months. The total amount of material was processed in three independent batches.

Subsequently, a brine solution was prepared using distilled water to achieve 3.75% sodium chloride and a pH of 4.0. For the experimental setup, 100 g of olives were pitted and placed in a glass jar containing the prepared brine. Both olives and brine were pasteurized at 80 °C for 20 min to control the development of vegetative forms of microorganisms. The olives were then inoculated under sterile conditions with different isolated and identified bacteria [23]: *Escherichia coli* CECT 4267, *Bacillus cereus* CECT 131, *Staphylococcus aureus* ATCC 25923, *Staphylococcus aureus* CECT 976, *Enterobacter cloacae* 42 (collection of the CAMIALI group, University of Extremadura) and *Bacillus subtilis* CECT 356. These strains were selected based on their significance as foodborne pathogens and hygiene indicators, allowing for a robust assessment of the product’s safety margins against accidental post-fermentation contamination.

Bacterial suspensions were appropriately diluted to achieve an inoculation concentration of 10^5^ CFU/mL in the brine solution. Uninoculated control olives were also prepared. In total, seven treatments were considered: six bacterial strains plus a Control. All treatments were performed in triplicate (biological replicates). Finally, olives were stored under controlled incubation conditions at room temperature (18 °C) for 30 days.

### 2.2. Physicochemical Characterization

Samples were analyzed after incubation to confirm the stability of the identified VOC. All measurements (except those described in Section 2.2.3 and Section 2.2.5) were performed in uniplicate on three independent samples (biological replicates) for each treatment, and the results were expressed as mean values.

#### 2.2.1. Assessment of Instrumental Color, Texture, and pH

Instrumental color was measured using the CIELAB color space, where L* represents lightness, a* denotes the red/green coordinate, and b* indicates the yellow/blue coordinate. Measurements were performed on 10 g samples. Color measurements were carried out using a CR-410 Chroma Meter (Konica Minolta Inc., Tokyo, Japan), previously calibrated with a standard white reference (Yxy). The instrument enabled the evaluation of lightness and chromatic coordinates according to the CIELAB system, following the methodology described elsewhere [24,25].

The pH of the samples was measured after the processing stage. For this purpose, 10 g of olive samples were homogenized with 25 mL of distilled water using a T-18 Basis Ultra-Turrax^®^ homogenizer (IKA, Staufen, Germany). The homogenate was filtered, and the volume was adjusted to 100 mL with distilled water. The pH was then measured using a digital pH meter ( (Basic 20, Crison Instruments, Barcelona, Spain) [26].

Sample firmness was evaluated using a TA.TX2 texturometer (Stable Microsystems, Surrey, UK), according to the procedure described by Zhao et al. [27]. The analysis was performed under the following conditions: pre-test speed of 1.6 mm/s, trigger force of 30 g, test speed of 2.20 mm/s, and post-test speed of 12 mm/s, with a penetration distance of 10 mm.

#### 2.2.2. Total Reducing Capacity and Radical Scavenging Potential

Total reducing capacity (expressed as total phenolic equivalents) was determined using the Folin–Ciocalteu colorimetric method, following the procedure described by Schaide et al. [26]. Briefly, olives were homogenized with distilled water (1:10 *w*/*v*) to obtain a diluted extract. Then, 1 mL of the extract was mixed with 1 mL of Folin–Ciocalteu reagent (Panreac, Barcelona, Spain), followed by the addition of 1 mL of saturated sodium carbonate (Na_2_CO_3_) solution and 7 mL of distilled water. The mixture was vortexed for 1 min after each reagent addition and subsequently incubated at room temperature for 90 min. Absorbance was measured at 725 nm using a GENESYS™ 10 UV–Vis spectrophotometer (Thermo Scientific™, Waltham, MA, USA). Gallic acid (purity ≥ 99%, ExtraSynthese) was used as the calibration standard (0.001–1 mol/L; R^2^ = 0.9998). Results were expressed as grams of gallic acid equivalents per 100 g of sample (g GAE 100 g^−1^) and are referred to as phenolic equivalents for comparative purposes.

The antioxidant activity was additionally evaluated using the DPPH (2,2-diphenyl- 1-picrylhydrazyl) radical scavenging assay, following the method described by Franco et al. [28]. The decrease in absorbance caused by the reduction of the DPPH radical was measured spectrophotometrically, and the antioxidant capacity of the samples was expressed as percentage of DPPH inhibition. Briefly, a fixed volume of each sample extract was mixed with a 0.1 mM DPPH methanolic solution, and the mixture was incubated in the dark at room temperature for 30 min. Absorbance was then recorded at 517 nm using a UV–Vis spectrophotometer, with a control solution containing DPPH without sample used as a reference. The percentage of radical scavenging activity was calculated according to the following equation: DPPH inhibition (%) = [(A_control − A_sample)/A_control] × 100.

#### 2.2.3. Olfactory Evaluation Analysis

A sensory panel of eight experts from CICYTEX and University of Extremadura (Spain), trained according to IOC standards, evaluated the table olives using an ISO 8589 [29]-compliant protocol within individual tasting cabins. The sensory evaluation conducted in this study was approved by the Bioethics and Biosafety Committee of the University of Extremadura (Approval Code: 228/2024; Approval Date: 23 October 2024). The panel assessed the intensity and type of off-odors on a structured 0–10 scale. Olfactory evaluation results were recorded as average values, with data considered valid only if the coefficient of variation was below 20% [21]. For each evaluated parameter, a mean value was obtained from the assessments of eight experts, with one value corresponding to each biological replicate.

#### 2.2.4. Volatile Organic Compound Profiling by HS-SPME–GC–MS

Volatile compounds were extracted from 2.0 g of olive paste using headspace solid-phase microextraction (HS-SPME) with a PDMS/DVB fiber. Samples were placed in 20 mL glass vials, hermetically sealed with PTFE/silicone septa, and equilibrated for 30 min at 40 °C under constant agitation [21]. After equilibration, the SPME fiber was exposed to the headspace for 30 min and subsequently thermally desorbed for 30 min at 250 °C in the injector port of a Bruker Scion 456-GC triple quadrupole equipped with a DB-WAXETR capillary column (30 m × 0.25 mm i.d., 0.25 µm film thickness). The GC oven temperature program was as follows: initial temperature of 40 °C held for 5 min, increased to 200 °C at 5 °C/min, and held for 10 min. Helium was used as the carrier gas at a constant flow rate of 1.0 mL/min. The mass spectrometer operated in electron impact mode (70 eV), scanning from *m*/*z* 40 to 400. Identification of volatile compounds was achieved by comparing mass spectra with those in the NIST library and by comparison of retention indices with literature data. Identification of volatile compounds was carried out by mass spectra; the retention index of compounds was calculated based on the retention index of external standard normal alkanes (C7~C40, Sigma-Aldrich, EE.UU) (Kovats Retention Index, KRI), and the NIST standard reference database was used. Furthermore, the quantitative analysis of the volatile organic compounds detected was performed by area comparison using an internal standard (2-octanol) of known concentration. The concentration of each VOC was calculated based on the ratio between the peak area of the target compound and that of the internal standard, assuming similar response factors. Results were expressed as µg of compound per g of olive paste.

#### 2.2.5. Olfactory Profiling via Electronic Nose (E-Nose)

A portable E-nose device (39 mm × 33 mm) developed by the University of Extremadura was used to characterize the aroma profile of spoiled olives, as previously described by Sánchez et al. [21]. This home-developed E-nose comprises 11 sensing signals obtained from 11 metal oxide semiconductor (MOS) sensors integrated into four chips: BME680 (Bosch), SGP30 (Sensirion), CCS811, and iAQ-Core (ScioSense). These sensors are broadly sensitive to different classes of volatile compounds, including alcohols, aldehydes, ketones, organic acids, esters, hydrocarbons, and sulfur-containing compounds, which are commonly associated with microbial spoilage processes. The sensor array therefore provides a non-selective but highly responsive global fingerprint of the volatile composition of the samples.

Samples were placed in standard tasting glasses (4 olives + 5 mL of brine) and covered with a watch glass. The glasses were then positioned on a heating block at 25 °C. The E-nose was placed above the glass to record data at 1 s intervals, with measurements transmitted to a computer via a Bluetooth interface. Nine measurements were taken for each sample. The sensors responded to the volatile compounds present in the headspace of the brine through chemisorption processes, producing measurable changes in electrical resistance. The peak response values from each sensor were extracted to generate a volatile fingerprint for subsequent multivariate statistical analysis. For each biological replicate, a total of three measurements were recorded.

### 2.3. Data Processing and Chemometric Statistical Modeling

To determine whether different treatments caused significant olfactory changes in each type of table olive, a one-way ANOVA followed by Tukey’s multiple range test (post hoc) was performed at a significance level α of 0.05. The analysis was conducted using SPSS 18.0 software, and results for the different treatments were included as means ± standard deviations.

E-nose data were processed using MATLAB software (R2022b, MathWorks, Natick, MA, USA) and analyzed through chemometric techniques. Principal component analysis (PCA), an unsupervised method, was first applied to reduce data dimensionality and to explore sample grouping based on VOC profiles. Prior to analysis, all variables were mean-centered and scaled to unit variance (autoscaling) to account for differences in measurement scales [30].

Subsequently, partial least squares regression coupled with discriminant analysis (PLS-DA), a supervised method, was employed to classify olive samples according to bacterial alterations. The optimum number of latent variables (LVs) for model fitting was selected by minimizing the root mean square error of cross-validation (RMSECV) using a leave-one-out procedure, ensuring high predictive accuracy while avoiding overfitting. Separate PLS-DA models were trained to discriminate samples affected by different species as well as strains within the same species. Model performance was evaluated using confusion matrices generated from cross-validation predictions, and classification accuracy was calculated as the percentage of correctly predicted samples.

## 3. Results

### 3.1. Microbial Growth of Table Olive Altered

**Figure 1:**
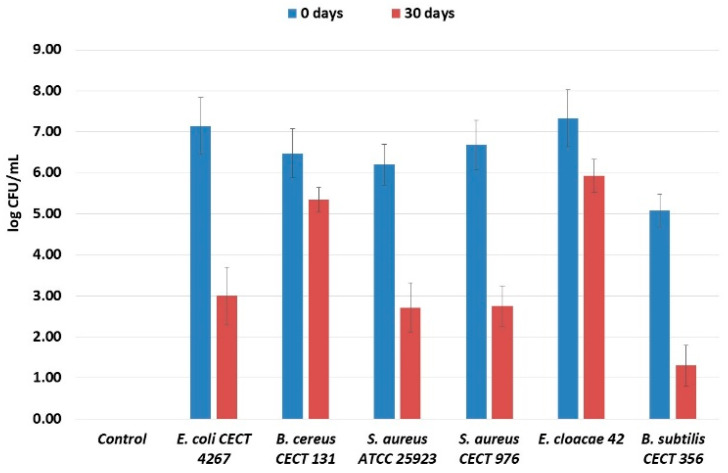
Microbial growth (log CFU/mL) of spoilage microorganisms inoculated in Spanish-style green olives at 0 days and after 30 days of incubation.

Microbial growth of spoilage microorganisms in Spanish-style green olives at the beginning of incubation (0 days) and after 30 days is shown in Figure 1. At day 0, all inoculated samples exhibited high initial microbial counts, ranging from approximately 5.1 to 7.3 log CFU/mL, depending on the microorganism. The highest initial populations were observed in olives inoculated with *E. cloacae* and *E. coli*, while *B. subtilis* showed comparatively lower initial counts.

After 30 days of incubation, a general reduction in microbial populations was observed for most spoilage microorganisms. The most pronounced decreases occurred in olives incubated with *B. subtilis, E. coli*, and both *Staphylococcus aureus* strains, whose counts dropped to values close to or below 3.0 log CFU/mL. In contrast, *B. cereus* and *E. cloacae* exhibited higher survival after 30 days, maintaining populations above 5.0 log CFU/mL, indicating greater tolerance to the conditions present in the table olives.

Overall, these results demonstrate that the table olives environment exerted a selective effect on spoilage microorganisms, resulting in strain-dependent survival patterns during incubation. *B. cereus* and *E. cloacae* appear to be more ecologically adapted to these conditions than transient, acid-sensitive contaminants such as *E. coli* and *S. aureus*.

### 3.2. Physicochemical Parameters of Altered Table Olive

The physicochemical characteristics, total phenolic content, and antioxidant activity of Spanish-style green olives inoculated with different bacterial strains are presented in Table 1. Significant differences (*p* < 0.05) were observed among treatments for most of the evaluated parameters. Each response variable was analyzed independently using a one-way ANOVA, with post hoc pairwise comparisons performed separately for each model using Tukey’s HSD test.

Lightness (L*) was significantly higher in the Control samples compared to all inoculated olives, which exhibited lower values, particularly those inoculated with *E. coli*, *S. aureus* ATCC 25923, *E. cloacae*, and *B. subtilis*. The a* coordinate increased in all inoculated samples relative to the Control, indicating a shift toward red tones, with the highest value observed in olives inoculated with *B. subtilis*. Conversely, b* values decreased significantly in inoculated samples, reflecting a reduction in yellow coloration. Chroma (C*) did not differ significantly among treatments, indicating that color saturation was not markedly affected by bacterial growth. However, hue angle (h) values differed significantly, with the Control showing the highest values and *B. subtilis* exhibiting the lowest hue angle, suggesting a perceptible shift in color tonality due to bacterial activity.

The Control olives exhibited the lowest pH value (3.95), whereas inoculated samples showed slightly higher pH values, ranging from 4.10 to 4.16, with significant differences among bacterial strains. Texture was significantly affected by microbial growth, with Control olives displaying the highest firmness. Inoculated samples generally exhibited lower texture values, particularly those inoculated with *E. coli*, *B. cereus*, and both *S. aureus* strains, while *E. cloacae* and *B. subtilis* inoculations result in intermediate firmness values.

Total phenolic content was significantly higher in the Control samples, followed by olives contaminated with *E. cloacae*. The lowest phenolic content was observed in olives inoculated with *S. aureus* CECT 976. These results indicate a strain-dependent effect on phenolic compound retention or degradation during incubation. Antioxidant activity varied significantly among the altered table olives. The highest antioxidant activity was observed in the Control samples, whereas olives inoculated with *B. cereus* and *B. subtilis* exhibited the lowest values. Intermediate antioxidant activity was recorded for olives inoculated with *S. aureus* strains and *E. cloacae*, suggesting that certain bacterial strains may enhance antioxidant capacity despite a reduction in total phenolic content.

### 3.3. Olfactory Evaluation

The results of sensory evaluation of Spanish-style green olives inoculated with different bacterial strains are presented in Table 2. Attributes were classified as positive or negative based on the intensity of predominant defect perception (PDP), according to the criteria established by the IOC [5]. Significant differences were observed among the different bacterial strains for each sensory attribute.

The Control treatment, consisting of not inoculated olives, exhibited prominent fruity and vinegary notes, with no negative attributes detected by the sensory panel. The intensity of these attributes ranged from 3 to 4 points on a 10-point scale, whereas negative attributes were not detected in the Control samples.

The olives inoculated with *B. cereus* and *S. aureus* ATTCC 25923 showed the lowest score for fruity attribute, representing a reduction of 55.6% compared to the Control. In contrast, olives inoculated with *E. coli* exhibited a slight increase in fruity perception relative to the Control.

The most prominent negative sensory attributes detected in table olives inoculated with bacterial strains were cooked and rancid notes, with intensities ranging from 0 to 2.5 on the sensory scale. Both defects showed comparable average scores across treatments. Olives inoculated with *B. subtilis* exhibited the highest intensity for the rancid attribute, whereas those inoculated with *B. cereus* showed the highest intensity for the cooked defect.

All inoculated samples exhibited at least one of these negative attributes, except for olives inoculated with *S. aureus* CECT 976, which did not show rancid characteristics. Wood defects were detected in approximately half of the inoculated samples (*B. cereus*, *S. aureus* ATTCC 252923 and *S. aureus* CECT 976), with *B. cereus* presenting the highest intensity (1.5).

Only one-third of inoculated samples exhibited chemical and humidity defects (*E. coli* and *E. cloacae* for chemical; *E. coli* and *B. cereus* for humidity). Among these, chemical defects were more pronounced than humidity-related ones, reaching an intensity score of 2. The least relevant negative attribute was acidity, which was only detected in olives inoculated with *B. subtilis*, with an intensity of 1.5.

Interestingly, all inoculated samples exhibited a sweet flavor, which was absent in the Control sample.

Overall, olives inoculated with *B. cereus* displayed the strongest negative profile, characterized by the highest intensities of cooked and wood defects and the greatest reduction in fruity perception. In contrast, olives inoculated with *S. aureus* CECT 976 exhibited the lowest levels of negative sensory attributes and the smallest impact on positive characteristics. In general, samples inoculated with *E. coli* and *B. cereus* showed more pronounced sensory deterioration, whereas those inoculated with *S. aureus* ATTCC 25293 and *S. aureus* CECT 976 presented fewer negative attributes.

### 3.4. Volatile Organic Compounds Analysis

**Figure 2:**
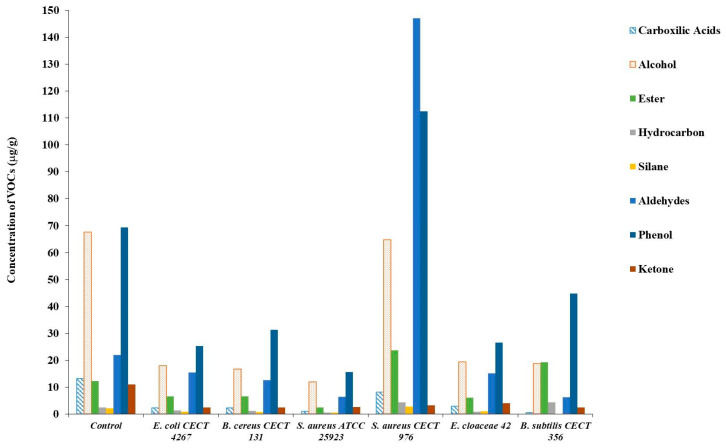
Distribution of VOC families (μg/g) in Spanish-style green olives contaminated with spoilage bacteria.

A total of 28 VOCs were identified and classified into eight chemical families: three phenols, ten alcohols, five aldehydes, four esters, three hydrocarbons, one carboxylic acid, one silane, and one ketone (Figure 2). Phenolic compounds represented the most abundant class across all samples, including the Control, followed by alcohols, whose relative abundance decreased in all inoculated samples compared with the Control.

Control olives exhibited a balanced volatile profile characterized by high levels of alcohols and phenols, along with moderate concentrations of esters and carboxylic acids. In contrast, olives inoculated with spoilage microorganisms showed marked alterations in their volatile composition. Samples inoculated with *E. coli*, *B. cereus*, and *S. aureus* ATCC 25293 displayed a general reduction in most volatile families, particularly carboxylic acids, ketones, and hydrocarbons.

A distinct volatile fingerprint was observed in olives inoculated with *S. aureus* CECT 976, which presented notably elevated levels of aldehydes and phenols, indicating a pronounced modification of the aromatic profile. Similarly, olives inoculated with *B. subtilis* showed increased concentration of ester and hydrocarbons compared with the other inoculated treatments, suggesting strain-specific metabolic activities influencing aroma formation.

The individual volatile compounds detected in Control olives and in olives inoculated with different spoilage bacteria are reported in Table 3.

Significant differences (*p* < 0.05) were observed for most volatile compounds, indicating a strong strain-dependent effect on the overall volatile profile. Alcohols constituted one of the predominant families in all samples. Control olives exhibited significantly higher concentrations of several alcohols, including 1-propanol, 3-methyl-1-butanol, (Z)-3-hexen-1-ol, and benzyl alcohol, which are typically associated with characteristic fermentation aromas. In contrast, olives contaminated with *E. coli*, *B. cereus*, and *S. aureus* ATCC 25923 showed a general decrease in alcohol content, whereas *E. cloacae* maintained or even increased the concentration of certain alcohols, such as 1-propanol, 1-heptanol, and benzyl alcohol.

Ester compounds were detected at relatively low levels in most samples; however, olives inoculated with *S. aureus* CECT 976 and *B. subtilis* exhibited markedly higher concentrations of n-propyl acetate, suggesting enhanced esterification activity associated with these strains [31]. n-Propyl acetate is characterized by solvent-like, fruity, and fusel odors [32], and may therefore contribute to the development of undesirable sensory attributes in table olives.

Aldehydes showed the most pronounced differences among treatments. Olives contaminated with *S. aureus* CECT 976 presented extremely high concentrations of aldehydes, particularly octanal and substituted benzaldehydes [21], indicating intense oxidative and degradative reactions. In contrast, aldehyde levels were considerably lower in the remaining treatments, including the Control.

Phenolic compounds, especially creosol and phenylethyl alcohol, were abundant in all samples but reached significantly higher levels in olives inoculated with *S. aureus* CECT 976. Intermediate phenolic concentrations were observed in *B. subtilis* and Control samples. Ketones and hydrocarbons were detected at lower concentrations and showed only moderate variations among treatments, with Control olives presenting the highest content of 6-methyl-5-hepten-2-one, while inoculated samples generally exhibited reduced levels.

### 3.5. Discrimination of Altered Table Olives Using the E-Nose

The potential application of E-nose technology for discriminating table olives intentionally spoiled with different bacterial strains was investigated. The E-nose system demonstrated a strong ability to detect subtle differences in the volatile profiles emitted by the samples, enabling discrimination among altered olives based on the response patterns generated by its sensor array.

**Figure 3:**
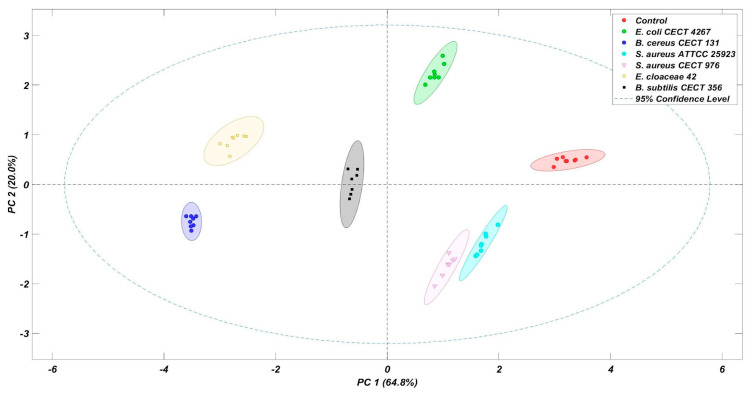
Score plot of the PCA of Spanish-style green olives inoculated with different bacterial strains.

The signals obtained from the E-nose, comprising eleven metal oxide sensors, were subjected to exploratory multivariate analysis using Principal Component Analysis (PCA) (Figure 3). The first principal component (PC1) accounted for 64.8% of the total variance, while the second principal component (PC2) explained an additional 20.0%. Together, these two components represented 84.8% of the total variability in the dataset, indicating that they were sufficient to capture the main sources of variation among the samples.

The score plots derived from PC1 and PC2 revealed a clear clustering of samples according to their spoilage status, evidencing the capacity of the E-nose system to discriminate between the spoiled and non-spoiled table olives based on their volatile fingerprints.

Further inspection of the score plots revealed that the samples were well separated along the first two principal components axes, with minimal overlap between groups. This observation confirms the robustness of the E-nose responses and the reliability of the measurements. Moreover, environmental conditions such as temperature and humidity were carefully controlled and remained consistent throughout the experiments, ensuring the stability of the sensor responses and the quality of the acquired data.

**Figure 4:**
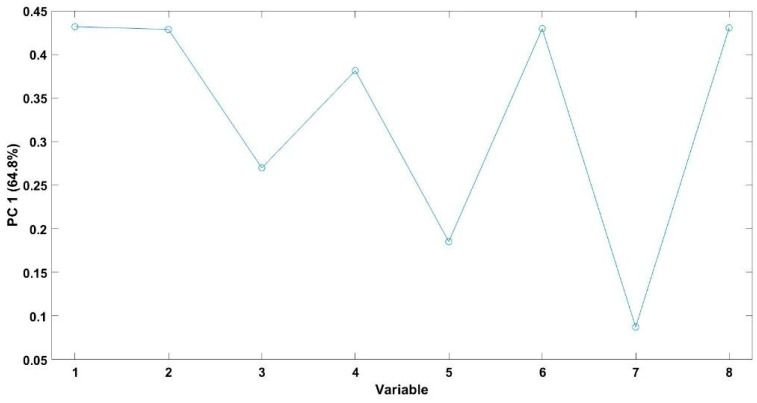
Loading plot of the PCA of Spanish-style green olives inoculated with different bacterial strains.

Figure 4 shows the loading plot corresponding to PC1, which provides insight into the contribution of each sensor to the observed discrimination. This analysis allowed differentiation among the spoilage microorganisms inoculated in Spanish-style green olives. Notably, all sensors exhibited a significant contribution to the discrimination process, suggesting that the overall volatile fingerprint, rather than any single compound or sensor, was responsible for the differentiation among samples. This result is consistent with the score plot shown in Figure 3, where the groups are clearly separated along the PC1 axis.

Given the satisfactory discrimination achieved through exploratory analysis, a supervised classification model was subsequently developed. Partial Least Squares regression coupled to Discriminant Analysis (PLS-DA) was applied to build a predictive model capable of classifying the samples according to their spoilage status. The performance of the model was evaluated using a leave-one-out cross-validation approach, and the resulting confusion matrix is shown in Table 4.

Given the multiclass nature of the problem (seven classes), model performance was evaluated based on overall classification accuracy rather than class-specific false positive or false negative rates. In the confusion matrix, the diagonal elements indicate the percentage of samples correctly classified for each class, and the sum of these diagonal values represents the overall prediction accuracy. Using this approach, nearly all samples were correctly classified, resulting in an overall accuracy of 90.4%.

These results are consistent with those obtained from the sensory panel evaluations and the volatile compound profiling, both of which also demonstrated clear differences among the altered olives. Altogether, these findings confirm the suitability and effectiveness of E-nose technology as a rapid, non-destructive tool for discriminating table olives altered by different microorganisms. This capability is attributed to the interaction between volatile compounds released by the samples and the sensor surfaces, where analytes are adsorbed and undergo oxidation reactions, leading to measurable changes in sensor resistance [33,34,35].

## 4. Discussion

The results of this study clearly demonstrate that bacterial spoilage of Spanish-style green table olives induces significant multivariate changes in the product, affecting microbial dynamics, physicochemical properties, sensory attributes, and, most importantly, the volatile fraction responsible for aroma. Within this context, electronic nose technology proved to be a highly effective tool for integrating these changes and discriminating spoiled samples based on their overall volatile fingerprint.

From a microbiological perspective, the table olives environment exerted a selective effect on the inoculated microorganisms, leading to strain-dependent survival patterns. While *B. subtilis*, *E. coli*, and *S. aureus* strains exhibited marked reductions in population after 30 days of incubation, *B. cereus* and *E. cloacae* maintained higher counts, reflecting greater tolerance to the table olives matrix. Although Romero-Gil et al. [36] reported *S. aureus* as the most resistant bacterium over a short period (48 h), compared with *E. coli* and other species, our 30-day study indicates that this resilience is transient. Over extended storage periods, the selective pressure of the olive environment is likely to inhibit such contaminants, favoring more ecologically adapted or spore-forming bacteria. In agreement with this, Argyri et al. [37] reported that *E. coli* could be detected until the 27th day in brine and the 19th day in olives. The strain-specific dynamics observed in our work are consistent with previous findings in fermented olives and other vegetable matrices, where microbial metabolism and survival strongly influence spoilage potential and metabolite production [38,39].

These microbial differences were directly reflected in physicochemical properties of the olives. Inoculated olives exhibited significant alterations in color, texture, pH, phenolic content, and antioxidant activity compared to the Control. The observed reduction in firmness, the increase in pH, and lower phenolic content in most inoculated samples suggest microbial degradation of structural and bioactive compounds, corroborating previous studies in which bacterial contamination accelerated matrix breakdown and diminished functional properties [21,31]. Such changes affect not only the technological quality of the product but also the release and transformation of volatile compounds, which ultimately govern sensory perception.

Sensory analysis further confirmed these effects. Olives inoculated with *B. cereus* and *E. coli* exhibited the highest intensity of negative attributes, including cooked, rancid, and woody notes, accompanied by a marked reduction in fruity aroma. In contrast, *S. aureus* CECT 976 induced milder sensory defects. These observations are consistent with previous reports indicating that specific spoilage bacteria in fermented olives can generate undesirable volatile compounds that dominate the sensory profile, often through enzymatic hydrolysis and oxidative reactions [32].

VOCs analysis revealed substantial alterations in aroma profiles, with inoculated olives generally exhibiting reduced alcohols and carboxylic acids, alongside strain-dependent increases in aldehydes, phenols, and esters. The exceptionally high concentration of aldehyde and phenols observed in olives inoculated with *S. aureus* CECT 976 indicates intense oxidative and degradative reactions, consistent with previous studies identifying aldehydes as key markers of spoilage in olives [21,35]. The elevated ester concentrations in *B. subtilis* and *S. aureus* CECT 976 inoculations suggest strain-specific metabolic pathways influencing aroma formation, in agreement with Alvanoudi et al. [31], who highlighted the role of bacterial metabolism in ester production and aroma modulation.

Within this complex aromatic landscape, the E-nose demonstrated remarkable sensitivity and discriminative capability [40]. PCA explained 84.8% of the total variance with only two components, and score plots showed clear separation between spoiled and non-spoiled samples, confirming the robustness of the device in detecting subtle differences in volatile composition. The loading plot indicated that all sensors contributed substantially to discrimination, emphasizing that the E-nose responds to the overall volatile fingerprint rather than to individual compounds, a characteristic previously reported in studies on fermented olives and other food matrices [33,34].

The predictive PLS-DA model further highlighted the potential of the E-nose, achieving a classification accuracy of 90.4%. These results are consistent with previous studies demonstrating that E-nose technology can reliably discriminate altered olives, differentiate processing methods, and detect spoilage conditions [33,34,35]. The underlying mechanism is well established: volatile compounds interact with the sensor surfaces, where adsorption and oxidation reactions induce a change in sensor resistance, generating signals that capture the chemical complexity of the sample.

Taken together, these findings confirm that the E-nose is a rapid, non-destructive, and highly sensitive tool for detecting bacterial spoilage in table olives. It effectively integrates microbial, chemical, and sensory alterations into a single analytical response, positioning the E-nose as a promising technology for industrial applications, including quality control, early spoilage detection, and classification of table olives according to microbial and sensory quality. This approach complements conventional sensory and chemical analyses, offering a robust and efficient alternative for monitoring product quality.

## 5. Conclusions

Contaminant survival in table olives is strain-dependent and represents a significant food safety concern, providing valuable data to future risk assessments. This differential survival can be largely attributed to their intrinsic resistance of specific strains to the hostile olive matrix, characterized by low pH, high salinity, and the presence of antimicrobial phenolic compounds. Certain strains, such as *Bacillus cereus*, exhibit superior persistence due to their ability to form endospores, which confer high resistance to environmental stress. Similarly, *E. cloacae* shows greater tolerance to acidic and osmotic stress compared to the other tested bacteria, whereas more sensitive strains are rapidly inactivated under these multi-stress conditions.

*B. cereus* and *E. coli* produce the most pronounced microbiological, physicochemical, and sensory alterations, reflected in the volatile profile, leading to detectable negative aroma attributes. The volatile composition of Spanish-style green olives was strongly influenced by the type of spoilage microorganism, demonstrating a clear strain-dependent effect. Control olives exhibited a balanced aroma dominated by alcohols and phenolic compounds, whereas inoculated samples showed significant modifications. Aldehydes, particularly octanal in *S. aureus* CECT 976, were markedly increased, reflecting intense spoilage and oxidative reactions. Alcohol levels generally decreased in *E. coli*, *B. cereus*, and *S. aureus* ATCC 25923, while esters such as n-propyl acetate were enhanced in *S. aureus* CECT 976 and *B. subtilis*, contributing to off-odors. These results indicate that specific VOCs can serve as reliable markers of microbial spoilage and strain-specific metabolic activity.

The E-nose successfully discriminated spoiled olives by responding to the overall volatile fingerprint with high accuracy, confirming its suitability as a rapid, non-destructive tool for quality monitoring. This technology holds substantial potential for industrial applications, enabling early detection of spoilage, supporting product classification, and complementing conventional sensory and chemical analyses.

## Figures and Tables

**Table 1:** Physico-chemical analysis of Spanish-style green olives inoculated with different bacterial strains. Values are expressed as means ± standard deviations (n = 3). Different lowercase letters indicate significant differences among treatments for each parameter (Tukey’s HSD, *p* < 0.05; ns: non-significant).

	Control	*E. coli* CECT 4267	*B. cereus* CECT 131	*S. aureus* ATTCC 25923	*S. aureus* CECT 976	*E. cloacae* 42	*B. subtilis* CECT 356
L*	48.0 ± 1.0 a	44.4 ± 1.6 c	46.2 ± 1.2 b	45.5 ± 1.1 c	46.4 ± 1.3 b	45.4 ± 1.1 c	44.9 ± 1.3 c
a*	0.2 ± 0.0 e	1.1 ± 0.1 c	1.2 ± 0.1 b	0.9 ± 0.2 d	0.9 ± 0.3 d	0.8 ± 0.2 d	1.9 ± 0.4 a
b*	25.0 ± 1.1 a	23.6 ± 1.2 b	23.0 ± 1.2 c	22.7 ± 0.8 c	23.3 ± 1.4 c	22.5 ± 1.2 d	22.2 ± 0.9 d
C*	24.7 ± 1.2 ns	23.2 ± 1.2 ns	23.1 ± 1.1 ns	22.7 ± 0.8 ns	23.7 ± 0.7 ns	22.7 ± 1.2 ns	22.1 ± 0.9 ns
h	89.9 ± 1.2 a	87.4 ± 1.0 c	86.0 ± 1.5 d	88.2 ± 1.1 b	87.0 ± 1.1 c	87.4 ± 1.2 c	85.0 ± 0.8 e
pH	3.95 ± 0.0 a	4.14 ± 0.0 c	4.12 ± 0.0 c	4.1 ± 0.1 b	4.16 ± 0.0 b	4.10 ± 0.0 d	4.1 ± 0.2 c
Texture (N)	0.66 ± 0.0 a	0.4 ± 0.1 c	0.43 ± 0.0 c	0.4 ± 0.1 c	0.4 ± 0.1 c	0.50 ± 0.0 b	0.52 ± 0.0 b
Total phenols (g GAE 100 g^−1^)	155.0 ± 5.6 a	144.1 ± 4.1 c	143.7 ± 2.5 c	142.8 ± 2.1 c	135.9 ± 2.1 d	149.7 ± 2.5 b	145.3 ± 3.0 c
DPPH (% of inhibited)	39.9 ± 2.1 e	44.3 ± 1.3 d	50.7 ± 2.5 a	46.4 ± 2.2 c	46.7 ± 1.5 c	47.8 ± 2.2 b	50.0 ± 3.4 a

**Table 2:** Positive and negative sensory attributes perceived by the trained panel for Spanish-style green olives inoculated with spoilage microorganisms. Values are expressed as means ± standard deviations (n = 8). Different lowercase letters indicate significant differences among bacterial treatments for each sensory attribute (Tukey’s HSD, *p* < 0.05). n.d.: not-detected. ns: non-significant.

	Control	*E. coli* CECT 4267	*B. cereus* CECT 131	*S. aureus* ATTCC 25923	*S. aureus* CECT 976	*E. cloacae* 42	*B. subtilis* CECT 356
Positive attribute							
Fruity	4.0 ± 0.2 d	4.1 ± 0.5 d	1.5 ± 0.3 a	2.5 ± 0.2 a	3.0 ± 0.3 c	2.5 ± 0.3 b	1.5 ± 0.4 b
Vinegar	3.5 ± 0.4 a	n.d.	n.d.	n.d.	n.d.	n.d.	n.d.
Negative attribute							
Rancid	n.d.	2.1 ± 0.3 c	1.0 ± 0.4 b	0.5 ± 0.4 a	n.d.	1.0 ± 0.5 b	2.5 ± 0.5 d
Cooked	n.d.	1.0 ± 0.3 a	2.5 ± 0.4 b	1.0 ± 0.3 a	1.0 ± 0.4 a	1.0 ± 0.5 a	1.0 ± 0.5 a
Wood	n.d.	n.d.	1.5 ± 0.2 b	0.5 ± 0.3 a	0.5 ± 0.1 a	n.d.	n.d.
Sweet	n.d.	3.0 ± 0.2 d	2.5 ± 0.4 c	2.0 ± 0.3 b	1.0 ± 0.2 a	2.0 ± 0.5 b	1.0 ± 0.3 a
Chemical	n.d.	2.0 ± 0.3 ns	n.d.	n.d.	n.d.	2.0 ± 0.5 ns	n.d.
Humidity	n.d.	1.5 ± 0.2 ns	1.5 ± 0.4 ns	n.d.	n.d.	n.d.	n.d.
Acidity	n.d.	n.d.	n.d.	n.d.	n.d.	n.d.	1.5 ± 0.4

**Table 3:** Quantification of VOC (μg/g) in Spanish-style green olives obtained by GC-MS. Values are expressed as means ± standard deviations (n = 3). Different lowercase letters indicate significant differences among spoilage microorganisms of each VOC (Tukey’s HSD, *p* < 0.05). n.d.: not-detected.

		Control	*E. coli* CECT 4267	*B. cereus* CECT 131	*S. aureus* ATTCC 25923	*S. aureus* CECT 976	*E. cloaceae* 42	*B. subtilis* CECT 356
**IR**	**Carboxilic Acids**							
2.263	Acetic acid	13.2 ± 2.5 a	2.3 ± 0.4 c	2.3 ± 0.3 c	0.96 ± 0.0 d	8.12 ± 1.1 b	2.86 ± 0.4 b	0.46 ± 0.0 e
	**Alcohol**							
2.002	1-Propanol	8.4 ± 1.2 a	5.8 ± 0.9 b	2.8 ± 0.3 c	3.4 ± 0.5 c	10.0 ± 1.1 a	6.3 ± 0.9 b	2.5 ± 0.3 c
4.218	3-Methyl-1-butanol	6.2 ± 1.1 a	2.2 ± 0.5 b	1.8 ± 0.1 b	1.5 ± 0.3 c	6.4 ± 0.7 a	2.0 ± 0.1 b	1.3 ± 0.2 c
5.716	2,3-Butanediol	4.6 ± 0.3 a	0.3 ± 0.0 d	0.52 ± 0.0 c	0.18 ± 0.0 e	1.90 ± 0.1 b	0.13 ± 0.0 e	0.58 ± 0.0 c
8.862	(Z)-3-Hexen-1-ol	9.2 ± 0.5 a	1.72 ± 0.31 d	1.6 ± 0.2 d	0.90 ± 0.0 e	5.6 ± 0.3 b	2.0 ± 0.1 d	2.7 ± 0.0 c
9.584	1-Hexanol	5.12 ± 0.3 a	0.44 ± 0.0 c	0.40 ± 0.0 c	0.27 ± 0.0 d	1.45 ± 0.1 b	0.54 ± 0.0 c	0.52 ± 0.0 c
14.361	4-Methyl-1-(1-methylethyl) cyclohexanol	3.01 ± 0.2 b	1.2 ± 0.3 c	1.2 ± 0.5 c	0.6 ± 0.0 d	6.5 ± 0.7 a	1.4 ± 0.1 a	n.d.
14.746	1-Heptanol	5.0 ± 0.5 b	0.99 ± 0.0 e	1.8 ± 0.1 c	1.5 ± 0.2 d	9.1 ± 1.0 a	1.3 ± 0.5 d	0.5 ± 0.0 f
16.689	Acetate-4-hexen-1-ol	7.2 ± 0.4 a	n.d.	n.d.	n.d.	n.d.	n.d.	3.1 ± 0.2 b
17.922	Benzyl alcohol	15.0 ± 1.8 b	4.2 ± 0.7 c	5.4 ± 0.5 c	2.9 ± 0.6 d	19.6 ± 1.5 a	4.6 ± 0.8 c	6.6 ± 0.6 c
19.965	1-Octanol	4.2 ± 0.5 a	1.0 ± 0.1 c	1.2 ± 0.1 b	0.7 ± 0.1 c	4.3 ± 0.3 a	1.2 ± 0.1 b	1.2 ± 0.1 b
	**Ester**							
3.750	n-Propyl acetate	2.8 ± 0.3 d	5.8 ± 0.6 c	6.0 ± 0.2 c	2.2 ± 0.1 d	21.5 ± 4.6 a	n.d.	19.3 ± 2.1 b
3.755	3-(Acetylthio)-2-methyl-propanoic acid	n.d.	n.d.	n.d.	n.d.	n.d.	5.4 ± 0.2	n.d.
6.871	Propyl ester of propanoic acid	7.6 ± 0.6 a	0.3 ± 0.0 c	0.29 ± 0.0 c	0.10 ± 0.0 d	1.0 ± 0.1 b	0.2 ± 0.0 d	n.d.
8.862	2-Hydroxy-ethyl ester-propanoic acid	1.8 ± 0.1 a	0.41 ± 0.0 c	0.34 ± 0.0 c	0.22 ± 0.0 d	1.2 ± 0.2 b	0.46 ± 0.0 c	n.d.
	**Hydrocarbon**							
7.242	2,4-Dimethyl-heptane	1.0 ± 0.1 a	n.d.	n.d.	n.d.	n.d.	n.d.	0.67 ± 0.0 b
19.363	1-Chloro-octane	n.d.	n.d.	n.d.	n.d.	n.d.	n.d.	0.68 ± 0.0
34.159	Copaene	1.4 ± 0.2 c	1.4 ± 0.1 c	1.21 ± 0.0 c	0.59 ± 0.0 e	4.4 ± 0.3 a	0.9 ± 0.1 d	3.0 ± 0.2 b
	**Aldehydes**							
13.958	Benzaldehyde	n.d.	1.2 ± 0.1 d	0.35 ± 0.0 f	0.74 ± 0.0 d	4.5 ± 0.2 a	1.5 ± 0.1 c	2.6 ± 0.2 b
16.391	Octanal	5.0 ± 0.3 b	1.11 ± 0.0 e	0.92 ± 0.0 e	0.72 ± 0.0 c	102.0 ± 5.9 a	1.2 ± 0.2 d	1.8 ± 0.2 c
18.381	Benzene acetaldehyde	11.0 ± 3.2 a	n.d.	n.d.	n.d.	n.d.	n.d.	0.6 ± 0.0 b
21.610	Nonanal	3.2 ± 0.3 b	2.7 ± 0.2 b	2.8 ± 0.3 b	1.3 ± 0.1 c	10.2 ± 2.3 a	2.7 ± 0.3 b	1.0 ± 0.1 d
22.606	2,5-bis[(trimethylsilyl)oxy]benzaldehydebis	3.1 ± 0.1 d	10.5 ± 1.2 b	8.4 ± 0.8 c	3.5 ± 0.3 d	30.3 ± 6.4 a	9.8 ± 1.1 c	0.25 ± 0.0 e
	**Phenol**							
20.740	2-Methoxy-phenol	4.0 ± 0.2 c	2.0 ± 0.0 f	3.4 ± 0.2 d	1.42 ± 0.0 g	12.3 ± 1.7 a	2.7 ± 0.2 e	5.2 ± 0.3 b
21.917	Phenylethyl Alcohol	8.4 ± 0.4 b	5.7 ± 0.5 d	6.4 ± 0.3 c	4.2 ± 0.2 e	23.0 ± 3.2 a	6.4 ± 0.3 c	7.4 ± 0.7 b
25.879	Creosol	57.0 ± 3.7 b	17.5 ± 2.1 e	21.5 ± 5.2 d	9.9 ± 1.1 f	77.1 ± 5.9 a	17.3 ± 6.3 e	32.3 ± 3.2 c
	**Ketone**							
15.590	6-Methyl-5-hepten-2-one	11.2 ± 2.5 a	2.5 ± 0.2 d	2.5 ± 0.3 d	2.5 ± 0.2 d	3.3 ± 0.2 c	4.00 ± 0.41 b	2.4 ± 0.1 d

**Table 4:** Confusion matrix obtained for a PLS-DA model for Spanish-style green olives inoculated with different bacterial strains. Values are expressed in percentages.

Real Class	Control	*E. coli* CECT 4267	*B. cereus* CECT 131	*S. aureus* ATTCC 25923	*S. aureus* CECT 976	*E. cloacae* 42	*B. subtilis* CECT 356
Control	14.2	0	0	0	0	0	0
*E. coli* CECT 4267	0	14.2	0	0	0	0	0
*B. cereus* CECT 131	0	0	12.4	0	0	0	0
*S. aureus* ATTCC 25923	0	0	0	10.6	3.1	0	0
*S. aureus* CECT 976	0	0	0	3.1	10.6	0	0
*E. cloacae* 42	0	0	3.5	0	0	14.2	0
*B. subtilis* CECT 356	0	0	0	0	0	0	14.2

## Data Availability

The original contributions presented in this study are included in the article. Further inquiries can be directed to the corresponding author.
